# ErbB signaling in brain injury regeneration: Pathway interactions and therapeutic potential

**DOI:** 10.4103/NRR.NRR-D-25-00155

**Published:** 2025-07-05

**Authors:** Patricia Pérez-García, Nora Martínez-Gómez, Sonia Vázquez-de Górgolas, Andrea Chamorro-Francisco, Ricardo Pardillo-Díaz, Pedro Nunez-Abades, Carmen Castro, Livia Carrascal

**Affiliations:** 1 Department of Biomedicine, Biotechnology and Public Health, Division of Physiology, University of Cadiz, Cadiz, Spain; 2 Department of Physiology, University of Seville, Seville, Spain; 3 Biomedical Research and Innovation Institute of Cadiz (INiBICA), Cadiz, Spain

**Keywords:** adult neurogenesis, brain-derived neurotrophic factor (BDNF)/TrkB pathway, diterpenes, ErbB, gamma-aminobutyric acid (GABA) transmission, ischemia, neuregulin, neurogenesis, neuroinflammation, neuroprotection, neuroregeneration, Notch signaling, traumatic brain injury

## Abstract

The ErbB signaling network has recently emerged as a key modulator of central nervous system responses to injury. This review provides a comprehensive overview of ErbB receptors and their ligands, highlighting canonical and non-canonical signaling mechanisms relevant to brain damage. We explore how ErbB signaling is dynamically regulated following injury and how it orchestrates processes such as neuroinflammation, gliosis, and neural repair. Special attention is given to its interplay with other critical pathways, including Notch signaling, and its roles within adult neurogenic niches, where it modulates neural stem cell behavior in response to damage. Based on accumulating preclinical evidence, we propose two therapeutic strategies for targeting ErbB signaling in brain injury: (1) dampening neuroinflammation through ErbB inhibition and (2) promoting neuroprotection and neurogenesis via neuregulin-1-mediated activation. The first strategy is supported by studies, which demonstrate that inhibition of ErbB1 limits neuroinflammation and supports neural repair in preclinical models. The latter strategy is supported by emerging studies demonstrating the significant potential of novel protein kinase C activating diterpenes in modulating ErbB signaling pathways through the regulation of neuregulin-1 release. Diterpenes, by influencing the ErbB pathway, may uniquely bridge the gap between neuroprotection and regeneration. Their potential to modulate inflammation and promote pro-regenerative cellular environments positions them as promising tools in the development of targeted therapies. By dissecting these mechanisms, we aim to shed light on the translational potential of ErbB-targeted therapies and their capacity to enhance endogenous repair processes in the injured brain.

## Introduction

The ErbB signaling network is involved in regulating a variety of cellular processes that are essential for central nervous system physiology, adult neurogenesis, neuroinflammation, and the repair of brain tissue. This network comprises four receptor tyrosine kinases, ErbB1 (also known as EGFR), ErbB2, ErbB3, and ErbB4, which interact with a diverse array of ligands (Sabbah et al., 2020). The ErbB receptors operate through complex dimerization processes, often forming homo- or heterodimers, providing the pathway with flexibility and specificity in transmitting extracellular signals to intracellular machinery (Buonanno and Fischbach, 2001; Sabbah et al., 2020). The interactions receptor-ligand initiate a series of downstream signaling events that include the activation of phosphatidylinositol 3’-kinase (PI3K)/Akt, mitogen activated protein kinase (MAPK)/extracellular-signal-regulated kinase (Erk), and phospholipase Cγ (PLCγ) pathways, all of which, regulate key cellular behaviors (Pandya and Pillai, 2014; Pankratova et al., 2018).

**Figure 1:**
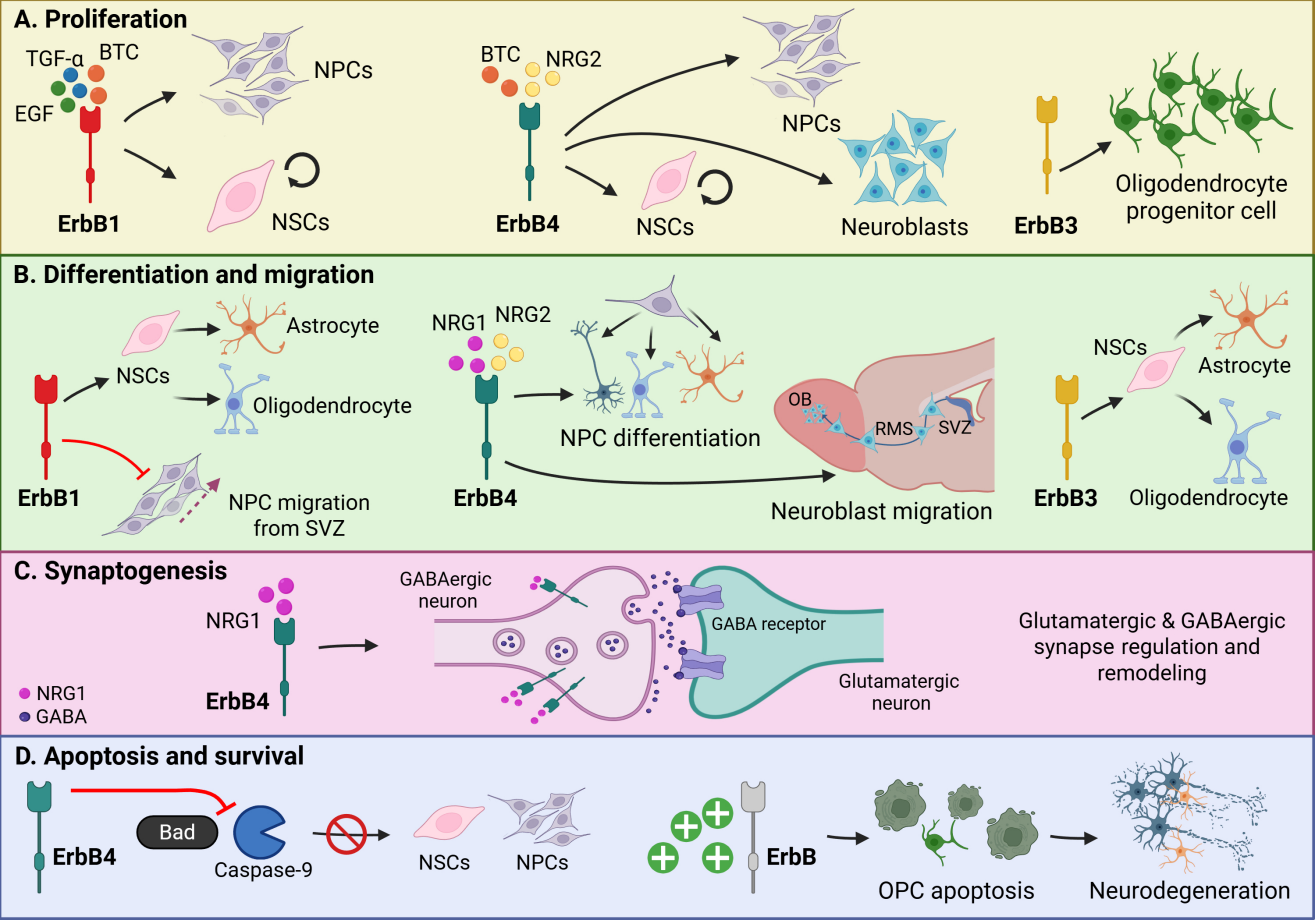
ErbB receptors in the adult brain: Regulators of neurogenesis, synaptogenesis, survival, and apoptosis. (A) Proliferation: ErbB1 activation by epidermal growth factor (EGF), transforming growth factor alpha (TGF-α), and betacellulin (BTC) drives the proliferation of neural stem cells (NSCs) and neural progenitor cells (NPCs). ErbB4, activated by neuregulin-2 (NRG2) and BTC, promotes the proliferation of NSCs, NPCs, and neuroblasts. ErbB3 signaling supports oligodendrocyte progenitor cell (OPC) proliferation. (B) Differentiation and migration: ErbB1 signaling facilitates NSC differentiation into astrocytes and oligodendrocytes (gliogenesis) and inhibits NPC migration from the subventricular zone (SVZ). ErbB4 activation via neuregulin-1 (NRG1) and NRG2 drives NPC differentiation into neurons (neurogenesis), astrocytes, and oligodendrocytes (gliogenesis). Additionally, ErbB4 promotes neuroblast migration from the SVZ along the rostral migratory stream (RMS) toward the olfactory bulb (OB). Also, ErbB3 signaling supports NSC differentiation into astrocytes and oligodendrocytes (gliogenesis). (C) Synaptogenesis: ErbB4 signaling supports synaptic maintenance and remodeling, ensuring proper synaptic connectivity, and regulates glutamatergic and GABAergic synapses. (D) Apoptosis and survival: ErbB4 signaling enhances cell survival by inhibiting the pro-apoptotic proteins Bad and Caspase-9, thus promoting NSC and NPC survival. Conversely, excessive ErbB receptor activation can induce OPC apoptosis and contribute to neurodegeneration. Created with BioRender.com.

Neurogenesis in the adult brain is a complex, hierarchical, and tightly regulated process that spans several stages, from the proliferation of neural stem cells (NSCs) and neural progenitor cells (NPCs) to their final integration into functional neural circuits. This process occurs in specific regions of the adult brain: one of particular interest in injury regeneration is the subventricular zone (SVZ), located adjacent to the striatal wall of the lateral ventricles, and another relevant neurogenic region is the subgranular zone (SGZ), found in the dentate gyrus of the hippocampus (Lim and Alvarez-Buylla, 2016; Obernier and Alvarez-Buylla, 2019; Kempermann et al., 2023; Gage, 2025a). These neurogenic niches harbor NSCs that give rise to new neurons and glial cells, contributing to brain plasticity, learning, and repair processes (Anton et al., 2004; Cope et al., 2020; Laham et al., 2024; Gage, 2025b). ErbB signaling, primarily through the activation of epidermal growth factor (EGF)–like ligands and neuregulins (NRGs), regulates the various phases of neurogenesis both during development and, more importantly, in the context of brain injuries in adulthood, orchestrating critical processes such as proliferation (**Figure 1A**), differentiation and migration (**Figure 1B**), synaptogenesis (**Figure 1C**) and cell survival (**Figure 1C**) and making it a key target for brain repair (Tagliaferro and Ponti, 2023).

Through activation by EGF-like ligands and NRGs, ErbB pathways stimulate the expansion of NSCs/NPCs in neurogenic niches, maintaining their self-renewal capacity and preventing premature differentiation (Romano and Bucci, 2020; Sabbah et al., 2020). This signaling also modulates lineage specification, guiding cell fate toward neuronal or glial phenotypes depending on contextual cues (Wee and Wang, 2017; Burgess, 2022). In migratory paths such as the rostral migratory stream, ErbB activity organizes neuroblast transit and ensures their proper integration into the target regions; its disruption leads to aberrant migration and impaired circuit formation (Anton et al., 2004; Mei and Nave, 2014). Furthermore, ErbB signaling plays a pivotal role in synaptic development and plasticity by regulating excitatory synapse formation, neurotransmitter receptor activity, and postsynaptic stability, especially in interneurons (Mei and Nave, 2014; Navarro-Gonzalez et al., 2021). Finally, ErbB activation enhances cell survival via PI3K/Akt signaling, counteracting apoptotic pathways and supporting long-term integration of newly generated cells, although dysregulated ErbB activity may contribute to pathological outcomes such as demyelination or cell loss (Hu et al., 2021).

The ErbB signaling pathway has recently gathered increasing attention due to its involvement in brain injury recovery, particularly in conditions such as ischemic stroke and traumatic brain injury (TBI) (Gu et al., 2017; Tang et al., 2018). Following brain injuries, ErbB signaling is often upregulated as a compensatory mechanism that promotes neuroprotection and tissue regeneration (Scafidi et al., 2014; Xue et al., 2019; Wang et al., 2023). In response to injury, different signals activating ErbB receptors (ErbB4 or ErbB1) can trigger opposing responses, either promoting or hindering the generation of new neurons (Aguirre et al., 2010; Geribaldi-Doldán et al., 2018; Sabbah et al., 2020; Cui et al., 2023; Gómez-Oliva et al., 2023; Groveman et al., 2023). Therefore, studying the role and balance of the ErbB pathway, its receptors, and ligands in the context of brain injuries is crucial to promote recovery and optimize therapeutic strategies aimed at enhancing neuroregeneration and reducing neurodegenerative outcomes.

The aim of this review is to provide a comprehensive analysis of the ErbB signaling pathway after brain injuries, such as ischemia and TBI. It will thoroughly explore recent advances in understanding the role of ErbB receptors and their ligands in neural repair mechanisms, examining how this pathway supports neuroregeneration, neuroprotection, and functional recovery. However, the review will also highlight the complexity of ErbB signaling, recognizing that its effects are not yet fully understood and may vary depending on the context, with activation leading to both protective and potentially detrimental outcomes. Additionally, the intricate crosstalk between ErbB and other pathways, such as brain-derived neurotrophic factor (BDNF)/TrK, GABAergic transmission, and Notch signaling, will be explored to assess how these interactions influence neurogenesis and tissue repair (Zhang et al., 2018; Deng et al., 2019; Piovesana et al., 2022). Emerging therapeutic strategies will be assessed in preclinical models, with a focus on their relevance and potential for clinical translation. By examining both the therapeutic potential and challenges of modulating ErbB signaling, this review aims to provide a nuanced perspective on its role in promoting neural regeneration, reducing inflammation, and optimizing recovery following brain injuries.

## Search Strategy

To focus on the role of ErbB signaling in brain injuries, particularly its interactions with other signaling pathways, such as BDNF, GABA, and Notch, as well as its therapeutic potential, we have conducted a comprehensive search using PubMed and Web of Science databases. Search terms included: “ErbB signaling,” “brain injury,” “neurogenesis,” “ischemia,” “traumatic brain injury,” “BDNF signaling,” “GABA transmission,” “Notch signaling,” “MAPK/Erk,” “PI3K/Akt pathways,” “subventricular zone,” and “dentate gyrus.” The searches targeted articles published between 2020 and 2025, although older studies were included based on their pivotal contributions to the understanding of the ErbB pathway in neurogenesis and brain repair. The search was conducted between May 2024 and April 2025. We have included preclinical studies, clinical trials, and review articles, all of which were published in peer-reviewed journals. Studies were limited to those published in English. Studies with insufficient data, duplicate publications, or those focused on unrelated signaling pathways were excluded. Articles were managed using Mendeley to organize and remove duplicates, and all references were carefully reviewed for relevance and contribution to the field.

## Overview of the ErbB Signaling Network

**Figure 2:**
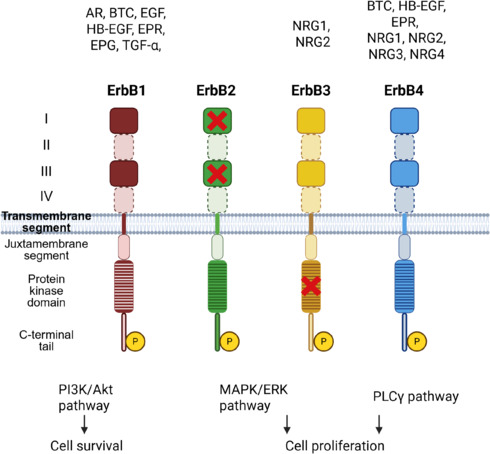
Structure of ErbB family receptors, their ligands, and signaling pathways. The receptors consist of an extracellular region with four domains (I–IV), a transmembrane segment, and an intracellular segment containing a protein kinase domain and a C-terminal tail. ErbB2 lacks a specific ligand, indicated by the crossing of domains I and III, while ErbB3 is kinase-impaired, as marked by the crossed protein kinase domain. Growth factor groups binding to each receptor are shown above them, and key downstream signaling pathways are depicted below. Created with BioRender.com. AR: Amphiregulin; BTC: betacellulin; EGF: epidermal growth factor; EPG: epigen; EPR: epiregulin; HB-EGF: heparin-binding epidermal growth factor-like growth factor; MAPK/Erk: mitogen-activated protein kinase/extracellular signal-regulated kinase; NRG: neuregulin; PI3K/Akt: phosphoinositide 3-kinase/protein kinase B (Akt); PLCγ: phospholipase C gamma; TGF-α: transforming growth factor alpha.

The ErbB receptor family comprises four transmembrane tyrosine kinase receptors: ErbB1, ErbB2, ErbB3, and ErbB4 (Yarden and Sliwkowski, 2001; Herbst, 2004). As illustrated in **Figure 2**, these receptors share a conserved structural organization, including an extracellular ligand-binding domain, a single transmembrane helix, and an intracellular region with tyrosine kinase activity (Herbst, 2004). The extracellular domain consists of four subdomains (I–IV); subdomains I and III are leucine-rich and mediate ligand binding, whereas subdomains II and IV are cysteine-rich and involved in receptor homo- or heterodimerization (Lee et al., 2006). While ErbB1, ErbB3, and ErbB4 are capable of binding specific ligands, ErbB2 lacks a known ligand and functions predominantly as a heterodimerization partner (Herbst, 2004). All ErbB receptors share a hydrophobic transmembrane segment anchoring them to the plasma membrane. The intracellular region comprises a juxtamembrane segment, a protein kinase domain, and a carboxyterminal tail containing multiple tyrosine residues subject to phosphorylation by the kinase domain (Lee et al., 2006; Burgess, 2022). Notably, ErbB3 possesses a catalytically impaired kinase domain, requiring dimerization with other family members to initiate downstream signaling (Yarden and Sliwkowski, 2001; Bertelsen and Stang, 2014). The ligand specificity for each receptor within the ErbB family is summarized in **Figure 2**.

ErbB ligands are initially synthesized as membrane-bound pro-ligands, consisting of extracellular, transmembrane, and cytoplasmic domains. To activate receptor signaling, the extracellular portion must be cleaved and released into the extracellular space (Sabbah et al., 2020). This proteolytic process is mainly mediated by ADAM17, a key member of the ADAM (A Disintegrin And Metalloprotease) family, which is responsible for the cleavage of several ErbB ligands, including heparin-binding EGF-like growth factor (HB-EGF), transforming growth factor alpha (TGF-α), and NRG1 (Blobel, 2005; Calligaris et al., 2021). The specificity of ADAM17 is regulated by phosphorylation of the pro-ligand’s cytoplasmic domain, a modification catalyzed by kinases of the protein kinase C (PKC) family (Dang et al., 2013). PKC enzymes are grouped into conventional (cPKC), novel (nPKC), and atypical (aPKC) isoforms (García-Bernal et al., 2018). For example, activation of cPKCs such as PKCα promotes the phosphorylation and ADAM17-mediated release of TGF-α and HB-EGF, while nPKCs such as PKCδ mediate the shedding of NRG1 (Dang et al., 2013).

The functional roles of the ErbB family ligands in central nervous system (CNS) are summarized below:

Amphiregulin binding to ErbB1 (Burgess, 2022) regulates proliferation, apoptosis, and migration in different cell types, playing an important role in controlling astrogliosis during CNS injuries that trigger neuroinflammation (Ito et al., 2019; Liston et al., 2022).

Betacellulin primarily binds to ErbB1 and ErbB4 and plays a role in adult neurogenesis by regulating NSC and NPC behavior. It contributes to the maintenance of these cells within neurogenic niches, regulating proliferation and differentiation (Zhang et al., 2018a; Wang et al., 2021).

EGF binds to ErbB1 and acts as a mitogen, regulating cell proliferation, differentiation, and tissue regeneration across multiple organs (Tito et al., 2025). EGF contributes to neurogenesis by promoting the expansion of NSC and NPCs and modulating their fate during development and in response to injuries (Torroglosa et al., 2007; Tito et al., 2025). EGF is also important in promoting the proliferation of oligodendrocyte precursor cells in the developing human cortex (Huang et al., 2020).

HB-EGF binds to ErbB1 and ErbB4, and contributes to CNS homeostasis by promoting cell survival, anti-inflammatory responses, and tissue protection. It is expressed in astrocytes and NSCs/NPCs and is upregulated in response to injury or inflammation (Cigliola et al., 2023; Adrain and Badenes, 2024; Linnerbauer et al., 2024; Moradi et al., 2024; Pastor-Alonso et al., 2024).

Epigen, which binds primarily to ErbB1, has limited but emerging relevance in the nervous system. Its physiological role in neural tissues appears minor under normal conditions. However, overexpression of epigen has been associated with peripheral neuropathy in animal models, leading to demyelination and axonal degeneration in the peripheral nervous system (Dahlhoff et al., 2013).

Epiregulin, a ligand for ErbB1 and ErbB4, promotes the proliferation of basal NPCs during neocortical development, contributing to cortical expansion in primates (Cubillos et al., 2024; Fernández and Borrell, 2024). In the adult nervous system, enhances nociceptive signaling, linking it to neuroinflammatory responses and chronic pain mechanisms (Martin et al., 2017).

TGF-α binds to ErbB1 and plays a key role in developmental and adult neurogenesis (Cesetti et al., 2009; Romano and Bucci, 2020), promoting proliferation and self-renewal of NSCs and NPCs, particularly in the SVZ and hippocampal dentate gyrus (Romero-Grimaldi et al., 2011; Gómez-Oliva et al., 2023b, 2024). In addition, in differentiating NPCs expressing EGFR, TGF-α is sufficient to induce preferential differentiation toward the glial lineage, while impairing the generation of new neurons (Romero-Grimaldi et al., 2011; Geribaldi-Doldán et al., 2018).

NRGs classified into four isoforms — NRG1, NRG2, NRG3, and NRG4 — are key regulators of nervous system development and repair, acting mainly through ErbB3 and ErbB4 receptors (Yarden and Sliwkowski, 2001). They promote Schwann cell and oligodendrocyte development (Birchmeier and Nave, 2008) and influence neuroblast and interneuron migration and differentiation (Tagliaferro and Ponti, 2023) as well as control synapse maturation and plasticity (Li et al., 2007; Exposito-Alonso et al., 2020; Rahman-Enyart et al., 2020) being relevant in neuropsychiatric disorders (Mei and Nave, 2014). Among the NRG family, the most relevant and extensively studied member is NRG1, which has a significant impact on peripheral and CNS injuries and repair (Alizadeh et al., 2018; Kataria et al., 2019; Xue et al., 2019; Ou et al., 2021; Cui et al., 2023a).

### Signaling mechanisms

The binding of an ErbB receptor to its ligand triggers a conformational change that enhances its affinity for another ErbB molecule, leading to homo- or heterodimerization (Olayioye et al., 1998). This activates the tyrosine kinase domain, which phosphorylates tyrosine residues in the C-terminal tail and initiates downstream signaling cascades (Yarden and Sliwkowski, 2001; Burgess, 2022). As shown in **Figure 2**, the three main pathways activated by ErbB receptors are PI3K/protein kinase B (Akt), PLCγ, and MAPK/Erk (Roskoski, 2014). The specific dimer formed determines ligand binding and selectively activates one of these pathways, contributing to diverse cellular responses (Yarden and Pines, 2012).

In the PI3K/Akt pathway, phosphorylated tyrosine residues recruit PI3K, which converts phosphatidylinositol 4,5-bisphosphate (PIP2) into phosphatidylinositol 3,4,5-trisphosphate (PIP3), a second messenger that activates Akt. Akt is fully activated by phosphoinositide-dependent protein kinase 1 and mammalian target of rapamycin complex 2 (mTORC2) (Fruman et al., 2017), leading to inhibition of pro-apoptotic proteins like Bad and stimulation of protein synthesis via the mTOR pathway. This signaling is key in regulating metabolism and promoting tumorigenesis (Hemmings and Restuccia, 2012).

In the PLCγ pathway, PLCγ binds phosphorylated tyrosines via its SH domain and becomes activated. It hydrolyzes PIP2 into inositol 1,4,5-trisphosphate (IP3) and diacylglycerol (DAG). IP3 releases calcium from the endoplasmic reticulum, and together with DAG, activates PKC, which phosphorylates downstream targets involved in cell survival and proliferation (Olayioye et al., 1998; Newton, 2010; Berridge, 2016).

In the MAPK/Erk pathway, adaptor proteins such as Grb2 bind to phosphorylated residues via their SH2 domain and recruit SOS, a guanine nucleotide exchange factor, which activates Ras by converting Ras-GDP to Ras-GTP (Yarden and Sliwkowski, 2001). Active Ras triggers a phosphorylation cascade: Raf activates MEK1/2, which then activates extracellular signal-regulated kinases (Erk1/2) (Paniagua et al., 2022). Erk1/2 enters the nucleus to phosphorylate transcription factors, promoting gene expression linked to cell cycle progression, proliferation, and differentiation (Olayioye et al., 1998; Shaul and Seger, 2007; Rabaneda et al., 2016).

## ErbB Signaling in Response to Brain Injuries

**Figure 3:**
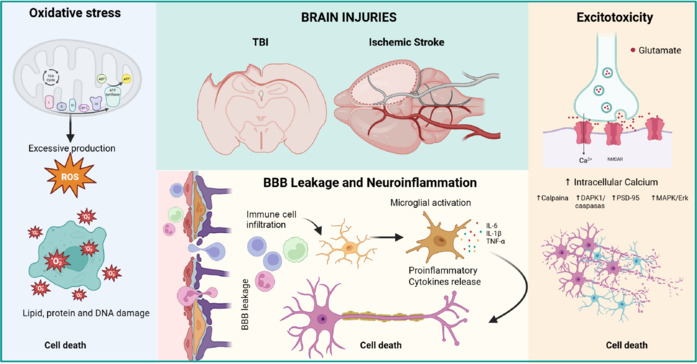
Shared mechanisms of brain injury in traumatic brain injury (TBI) and stroke: Excitotoxicity, neuroinflammation, and oxidative stress. The figure illustrates the interconnected pathological processes underlying TBI and ischemic stroke. Oxidative stress: Mitochondrial dysfunction leads to excessive production of reactive oxygen species (ROS), resulting in lipid, protein, and DNA damage, ultimately causing cell death. Blood–brain barrier (BBB) leakage and neuroinflammation: Damage to the BBB allows immune cell infiltration, triggering microglial activation and the release of pro-inflammatory cytokines, including interleukin (IL)-6, IL-1β, and tumor necrosis factor-alpha (TNF-α), which exacerbate neuroinflammation and neuronal damage. Excitotoxicity: Increased glutamate release leads to overactivation of NMDA receptors, elevated intracellular calcium levels, and activation of downstream pathways (e.g., calpain, DAPK1, postsynaptic density protein 95 (PSD-95), and MAPK/Erk), culminating in synaptic dysfunction and neuronal death. Created with BioRender.com.

**Figure 4:**
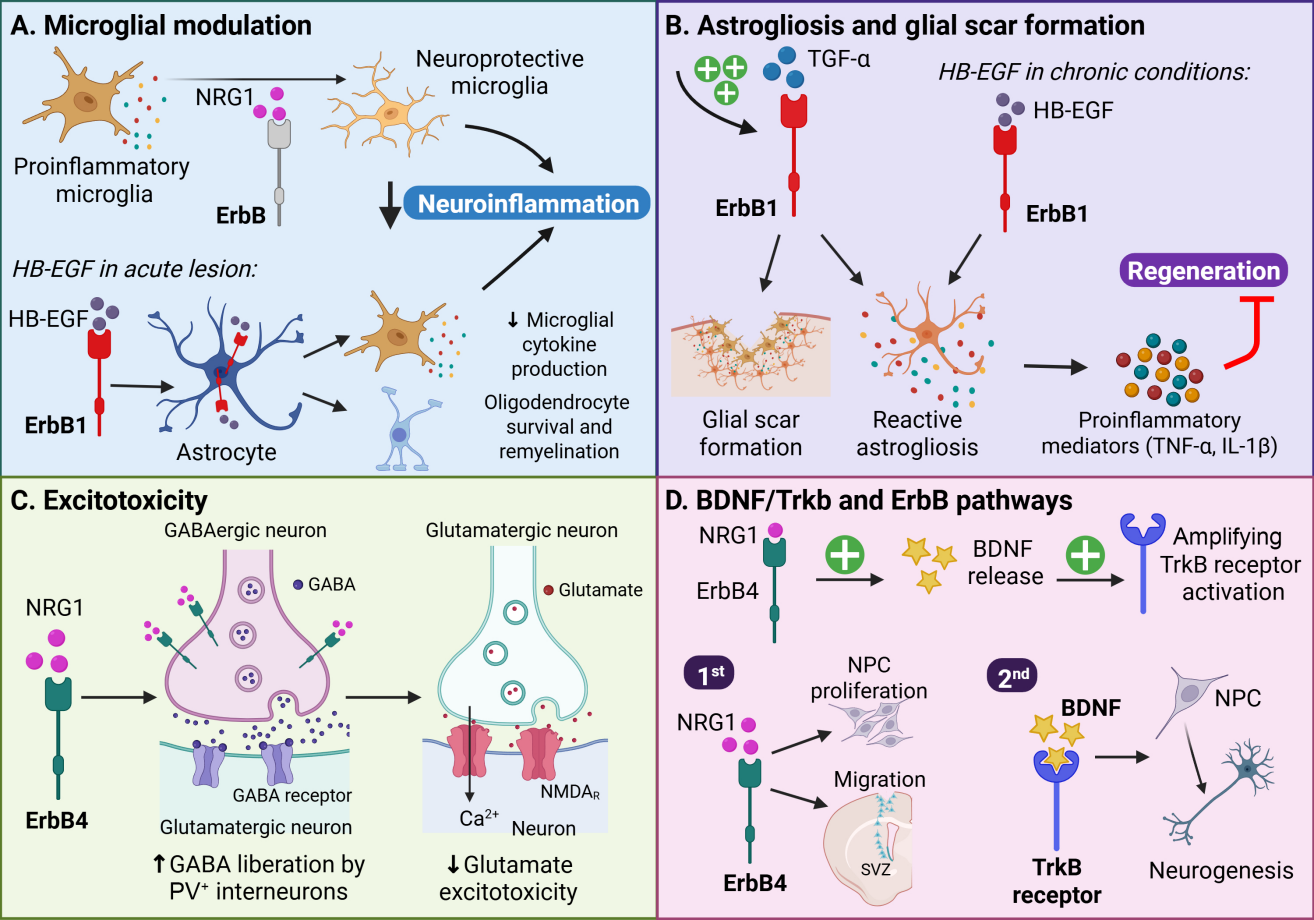
ErbB receptor signaling in brain injury: Mechanisms of neurorepair, neuroprotection, and gliosis. (A) Microglial activation: Neuregulin-1 (NRG1) signaling via ErbB receptors shifts pro-inflammatory microglia to a neuroprotective phenotype, reducing inflammation. Blocking ErbB2 signaling decreases microglial proliferation and activation, further mitigating inflammation. (B) Glial scar formation: ErbB1 activation, upregulated by transforming growth factor alpha (TGF-α), drives reactive astrogliosis and glial scar formation. This process triggers the release of pro-inflammatory mediators, exacerbating neuroinflammation. (C) Excitotoxicity: ErbB4 activation by NRG1 increases gamma-aminobutyric acid (GABA) release from parvalbumin positive (PV^+^) interneurons, reducing glutamate-induced excitotoxicity by lowering glutamate release and intracellular calcium levels. (D) Brain-derived neurotrophic factor (BDNF)/Tropomyosin receptor kinase B (TrkB) and ErbB pathways: ErbB4 activation by NRG1 boosts BDNF release, which stimulates TrkB phosphorylation. Additionally, NRG1 signaling through ErbB4 initiates neural progenitor cells (NPC) proliferation and migration. Subsequently, BDNF signaling via TrkB promotes NPC differentiation into neurons, supporting neurogenesis. Created with BioRender.com.

Brain injuries trigger a cascade of neurodegenerative events, including excitotoxicity, oxidative stress, and neuroinflammation (**Figure 3**), which lead to secondary injury processes that last from hours to years (Simon et al., 2017; Manivannan et al., 2021; Alsbrook et al., 2023; Neves et al., 2023). The expression of ErbB receptors, primarily ErbB4 and ErbB1, and their ligands is modulated in brain injuries, where these receptors are involved in neuroprotection, modulation of neuroinflammation, and the promotion of repair mechanisms (**Figure 4**; Thapa et al., 2021; Gómez-Oliva et al., 2023a; Noll et al., 2024). Particularly, TBI or ischemic injuries induce the expression of NRG1 (Pardillo-Díaz et al., 2025) and TGF-α (Romero-Grimaldi et al., 2011; Gómez-Oliva et al., 2023a) and EGF (Sun et al., 2010; Tang et al., 2020) whereas other types of injury, such as multiple sclerosis or seizures, trigger the additional release of HB-EGF.

### ErbB and neuroinflammation in response to brain injuries

#### Role of NRG1-ErB4

Microglia are critical players in neuroinflammation and the innate immune response during various neuropathological conditions (dos Santos et al., 2021; Jung et al., 2025). In response to injury, microglia quickly activate and adopt different phenotypes, depending on the nature of the damage (dos Santos et al., 2021; Henry and Loane, 2025). Proinflammatory microglia enhance the production of cytokines and mediators, which drive neuroinflammation and contribute to tissue damage whereas proregenerative microglia release anti-inflammatory factors, which help resolve inflammation and promote the clearing of tissue debris during the repair process (Quan and Zhang, 2023; Zhou et al., 2025). These findings highlight the diverse and essential roles that microglia play in the brain’s innate repair mechanisms following injury or disease. NRG1-ErbB4 signaling influences the activation of microglia (Mei and Nave, 2014; Ma et al., 2022). Under normal conditions, microglia act as an active sensor that scans the brain for signs of damage or abnormalities (Kettenmann et al., 2011). In response to CNS damage, microglial activation facilitates the removal of cellular debris, pathogens, and cellular remnants resulting from injury or disease (Marín-Teva et al., 2011). However, prolonged microglial activation may lead to a pro-inflammatory state that damages tissue and contributes to glial scar formation, impeding axonal regeneration and neuronal repair (dos Santos et al., 2021). Interestingly, as shown in **Figure 4A**, NRG1 modulates the microglial response by shifting from a pro-inflammatory to a neuroprotective phenotype (Alizadeh et al., 2018). In addition to the established role of the NRG1-ErbB4 pathway in modulating microglial activity, this pathway suppresses the TLR4-NF-κB-NLRP3 inflammasome pathway, a major driver of neuroinflammation. As a result, levels of inflammatory markers such as interleukin-1β, apoptosis-associated speck-like protein containing a CARD, and cleaved caspase-1 are significantly reduced, leading to attenuation of the inflammatory response in the hippocampus (Liu et al., 2025).

#### Role of TGF-α-ErbB1

In response to TBI, ErbB1 ligands such as TGF-α appear within the perilesional area, mainly expressed by astrocytes and microglial cells. Since perilesional ErbB1 is mainly expressed in astrocytes, the release of ErbB1-activating molecules leads to astrogliosis (Romero-Grimaldi et al., 2011; Gómez-Oliva et al., 2023a) and glial scar formation (**Figure 4B**). Likewise, ErbB1 activation in ischemia, often triggered by TGF-α, could contribute to detrimental outcomes, including inflammation, oxidative stress, and neuronal damage and death (Tavassoly et al., 2020). The upregulation of ErbB1 occurs primarily in astrocytes, leading to reactive astrogliosis and glial scar formation, which physically impair repairing mechanisms (Pandya and Pillai, 2014; Zhang et al., 2016a; Wurzelmann et al., 2017; Gómez-Oliva et al., 2023a, 2024). The scar attracts microglial cells and together with astrocytes creates an environment rich in pro-inflammatory mediators and neurotoxic molecules such as tumor necrosis factor-alpha and interleukin-1 beta, contributing to secondary tissue damage, including further neuronal loss and impaired regeneration (Erschbamer et al., 2007; Li et al., 2011, 2014; Yang et al., 2011; Abrams et al., 2012; Zunke and Rose-John, 2017; Gómez-Oliva et al., 2023a; Wang et al., 2023).

#### Role of HB-EGF-ErbB1

HB-EGF is also an injury-induced growth factor that modulates neuroinflammatory responses after CNS damage by acting through ErbB1 receptor signaling pathways (Cigliola et al., 2023; Linnerbauer et al., 2024). Under hypoxic and acute CNS lesions, the expression of HB-EGF is induced in astrocytes, which in turn exerts anti-inflammatory effects by reducing microglial cytokine production and promoting both oligodendrocyte survival and remyelination. Together, these actions contribute to the resolution of neuroinflammation and the preservation of tissue integrity (Cigliola et al., 2023; Moradi et al., 2024). Although this regulatory mechanism predominates during early-stage inflammation — as observed in multiple sclerosis patients with clinically isolated syndrome, where elevated cerebrospinal fluid levels of HB-EGF correlate with a protective response — this role shifts in chronic or severe conditions. In such context, such as those triggered by seizures, HB-EGF behaves similarly to TGF-α, enhancing EGFR-dependent reactive astrogliosis and contributing to an exacerbated inflammatory environment (Pastor-Alonso et al., 2024). These findings underscore the dual context-dependent role of HB-EGF: while it supports resolution and repair in early inflammation, its persistent activation under pathological conditions can instead drive maladaptive gliosis and sustained neuroinflammation.

Overall, ErbB1 inhibition in severe injuries reduces astrogliosis and the size of the glial scar and facilitates repairing mechanisms (Gómez-Oliva et al., 2023a).

### Interactions of the ErbB signaling cascades with other signaling events in response to injuries

The effects of ErbB family signaling following brain injury are profoundly influenced by the interplay of distinct molecular pathways. Here, we explore these roles with a specific focus on their interactions with GABAergic transmission and BDNF/Tropomyosin receptor kinase B (TrkB) pathways, which collectively help guide repair processes and neurogenesis in neurogenic niches.

#### Protective role of ErbB4 activation against excitotoxicity in ischemia and traumatic brain injury via neuregulin-1-GABAergic signaling

The neuroprotective effects of NRG1-ErbB4 signaling in brain injuries are mediated in part by its regulation of GABAergic transmission.

In a physiological context, ErbB4 signaling plays a pivotal role in maintaining the balance between excitatory and inhibitory synaptic transmission, a critical determinant of neural circuit stability and cognitive function (Navarro-Gonzalez et al., 2021). ErbB4 is highly expressed in GABAergic neurons, particularly in parvalbumin-positive (PV^+^) interneurons within the cortex and hippocampus, where it regulates the formation and maintenance of excitatory synapses in these inhibitory cells (Wang et al., 2018, 2024). Activation of ErbB4 by NRG1 enhances postsynaptic density through the stabilization of scaffolding proteins such as postsynaptic density protein 95 and increases the number of excitatory synaptic contacts on interneurons (Krivosheya et al., 2008). This input is essential for modulating interneuron activity, which in turn regulates the overall inhibitory output onto principal excitatory neurons. An *in vivo* study further demonstrates that ErbB4 knockout in GABAergic interneurons leads to a significant reduction in excitatory synapses and dampened postsynaptic potentials, ultimately disrupting the inhibitory tone of neural networks (Wang et al., 2018). Moreover, ErbB4-mediated signaling contributes to synaptic plasticity by enhancing α-amino-3-hydroxy-5-methyl-4-isoxazolepropionic acid receptor-mediated currents in interneurons, supporting dynamic regulation of excitatory drive (Li et al., 2007; Chen et al., 2008). Thus, ErbB4 functions as a central modulator of excitatory inputs onto inhibitory neurons, ensuring proper excitatory/inhibitory balance, a fundamental property of healthy brain function.

In most brain injuries, balance is disrupted, typically skewed toward excessive excitatory input. In this context, NRG1-ErbB4 signaling becomes crucial, as it helps restore homeostasis by enhancing GABAergic interneuron function and reinforcing inhibitory control, reducing excitotoxicity (**Figure 4C**) and limiting neuronal death (Deng et al., 2019). In models of middle cerebral artery occlusion, activation of NRG1-ErbB4 signaling has been shown to improve neurobehavioral outcomes and reduce infarct size by enhancing the activity of PV^+^ interneurons (Neves et al., 2023). Likewise, the administration of NRG1 post-ischemia has been shown to reduce infarct volumes and restore GABAergic transmission, highlighting the therapeutic potential of targeting this pathway (Wang et al., 2018; Vullhorst et al., 2023). Likewise, GABAergic transmission is also enhanced in response to TBI as a compensatory mechanism to counteract excitotoxicity. Following TBI, both NRG1 and ErbB4 are upregulated in the injured cortex, with ErbB4 primarily expressed in PV^+^ interneurons. Conditional deletion of ErbB4 in these interneurons leads to increased lesion volume, neuronal loss, and neuroinflammation, indicating that ErbB4 signaling is essential for limiting damage. Conversely, intranasal administration of recombinant NRG1 enhances functional recovery and reduces glutamate-induced neurotoxicity, an effect abolished by ErbB4 inhibition or GABA receptor blockade. These findings support the idea that ErbB4 activation in GABAergic neurons mitigates excitotoxicity and contributes to neuroprotection following TBI (Deng et al., 2019).

Nonetheless, the upregulation and release of NRG1 do not always lead to pathway activation, as an imbalance between the ligand and receptor expression can occur. Thus, in models of neonatal hypoxic-ischemic injury, a significant increase in hippocampal NRG1 levels is observed shortly after the insult, presumably as a compensatory mechanism, yet this is insufficient to activate protective signaling due to a marked downregulation of ErbB4 expression and phosphorylation. This leads to impaired activation of downstream effectors such as Akt, diminished maturation and survival of PV^+^ interneurons, and persistent excitatory/inhibitory imbalance (Spahic et al., 2023). These findings suggest that, although NRG1 is upregulated after ischemia, the loss of ErbB4 receptors found in some studies impairs the system’s capacity to counteract excitotoxicity, further highlighting the importance of preserving ErbB4 integrity for effective neuroprotection.

#### Interaction between brain-derived neurotrophic factor/Tropomyosin receptor kinase B and ErbB pathways in brain injury

BDNF/TrkB and ErbB signaling pathways are critical for neurogenesis, neuroprotection, and repair following brain injuries such as TBI and ischemia. These pathways work independently and synergistically to support neuronal survival, differentiation, and tissue recovery. BDNF signaling via TrkB is crucial for neuronal survival, synaptic plasticity, and neurogenesis. Upon binding to TrkB, BDNF activates key signaling cascades, including IRS-1/PI3K/Akt, Ras/MAPK/Erk, and PLCγ/DAG/IP3, which collectively promote neuronal survival, growth, and differentiation (Wurzelmann et al., 2017; Zhu et al., 2021; Mojtabavi et al., 2022; Kodali et al., 2023). In the context of brain injuries, these pathways mitigate cell death, enhance synaptic plasticity, and facilitate the repair of damaged neurons (Wurzelmann et al., 2017; Abdelhamid et al., 2024; Tsimpolis et al., 2024). However, ischemic injury results in the downregulation of endogenous BDNF and its downstream signaling, which is unfavorable for neural repair (Li et al., 2020; Mojtabavi et al., 2022). Notably, individuals with higher serum BDNF levels post stroke show better outcomes and decreased risk of secondary damage (Zhu et al., 2023; Chang et al., 2024). Intranasal administration of BDNF restores signaling, including the phosphorylation of TrkB, Akt, PLCγ, Erk, and CREB, which collectively promote neuronal survival, neurogenesis, synaptic plasticity, and neuroblast migration to the lesion site. Evidence includes increased Ki67-positive cells (proliferating cells) and CD31-positive cells (angiogenesis markers) in the peri-infarct region (Zhou et al., 2023). Moreover, BDNF mitigates oxidative stress and inflammation, protecting neurons via pathways like Akt/CREB/BDNF. Treatments such as notoginsenoside R1, saxagliptin, or salidroside also activate these pathways, enhancing neurogenesis and promoting cognitive recovery (Zhu et al., 2021; Abdelhamid et al., 2024; You et al., 2024; Zheng et al., 2024).

ErbB signaling enhances BDNF-mediated neurogenesis and neuroprotection by amplifying TrkB receptor activation (**Figure 4D**). TBI and ischemia induce a robust inflammatory response that includes the release of NRGs (Wurzelmann et al., 2017). In response to NRG1 release, ErbB4 activation boosts BDNF release, which further promotes TrkB phosphorylation and downstream signaling, facilitating neuronal differentiation and synaptic restoration (Pandya and Pillai, 2014). ErbB4 activation by NRG1 enhances NPC proliferation and directs them to the damaged cortical regions. Once there, BDNF/TrkB ensures NPC differentiation into functional neurons rather than glial cells, thereby aiding tissue repair and functional recovery throw balancing neurogenesis and inflammation (Wurzelmann et al., 2017; Zhu et al., 2021; Tsimpolis et al., 2024). Moreover, TrkB phosphorylation is enhanced by ErbB4 activation, which further promotes neurogenesis in the areas surrounding the lesion, aiding in the recovery of synaptic connections and cognitive functions. This highlights the importance of maintaining a balance between ErbB and TrkB signaling to optimize recovery from brain injury (Wurzelmann et al., 2017).

Beyond its effects on modulating neuroinflammation, astrogliosis, and neuroprotection, ErbB receptor signaling plays a pivotal role in brain repair mechanisms. Adult neurogenesis and neuronal replacement in response to brain injuries are so profoundly influenced by these signaling events that they warrant detailed discussion in the paragraphs below.

## Adult Neurogenesis and Brain Damage: Role of ErbB Receptors

**Figure 5:**
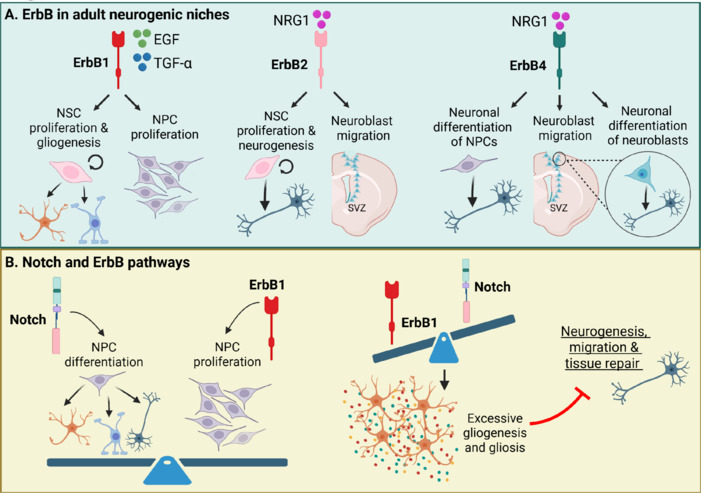
ErbB receptors and Notch signaling in neurogenic niches: Coordinated regulation of brain repair mechanisms. (A) Neurogenic niches: ErbB1 activation promotes the proliferation of neural stem cells (NSCs) and neural progenitor cells (NPCs), as well as gliogenesis. Neuregulin-1 (NRG1) signaling through ErbB2 enhances NSC proliferation, neurogenesis, neuroblast migration and cellular maintenance in the neurogenic niche. Additionally, ErbB4 activation promotes NSC proliferation, neurogenesis and NSC migration. (B) Notch and ErbB pathways: The interplay between Notch and ErbB1 signaling regulates NPC differentiation and NPC proliferation. Excessive ErbB1 activation disrupts this balance, leading to excessive glial formation and impaired repair mechanisms. Created with BioRender.com.

In response to brain injuries, adult neurogenesis is transiently activated in canonical neurogenic niches such as SVZ and SGZ. NSCs in these regions become reactive and proliferate in an attempt to repair the damaged tissue (**Figure 5A**; Llorens-Bobadilla et al., 2015; Jurkowski et al., 2020; Thapa et al., 2021; Geribaldi-Doldán et al., 2023). In the SVZ, NSCs activate and generate neuroblasts that migrate toward non-canonical regions such as the striatum and cortex, where some undergo differentiation and partial functional integration. In the SGZ, granule cell production increases transiently after injury, although a proportion of the newly generated neurons exhibit morphological and functional abnormalities (Ceanga et al., 2021). The promotion of adult neurogenesis in response to brain injuries is accompanied by significant upregulation of ErbB receptor signaling, particularly ErbB1, ErbB2, and ErbB4, each of which is stimulated by specific ligands and contributes uniquely to neurogenic responses post-injury (Romero-Grimaldi et al., 2011; Gómez-Oliva et al., 2023a, 2024).

ErbB1 is upregulated following TBI and stroke, particularly in the SVZ and SGZ regions (Romero-Grimaldi et al., 2011; Gómez-Oliva et al., 2023a, 2024), as well as the expression of its ligands EGF, TGF-α and HB-EGF. HB-EGF expression is induced by hypoxia/ischemia and has been shown to trigger the proliferation of NPCs. Jin et al. (2002) found that HB-EGF rises by approximately 50% in hypoxic cortical cultures and that adding HB-EGF significantly increased BrdU incorporation in EGFR-expressing neural cells. EGF infusion post-TBI enhances NSC and NPC proliferation, however, it skews differentiation toward astrocytes, limiting neurogenesis while promoting reactive gliogenesis. Despite this, EGF exerts neuroprotective effects by preserving hippocampal neurons in regions such as CA3 and improving cognitive performance, likely by enhancing glial support and mitigating excitotoxicity (Sun et al., 2010). TGF-α also promotes NSC/NPC proliferation and has been shown to induce preferential differentiation toward glial lineages over neurons when administered to differentiating NPCs expressing EGFR, further reinforcing the gliogenic bias of ErbB1 activation post-injury (Romero-Grimaldi et al., 2011).

Conversely, the blockage of the Erb1 receptor during the acute phase of TBI or stroke has been shown to reduce gliosis and apoptosis and enhance neuronal differentiation, facilitating improved functional recovery (Torroglosa et al., 2007; Nakano et al., 2023; Gómez-Oliva et al., 2024).

Thus, ErbB1 signaling has a dual role after brain injury in the neurogenic niches. It is essential for activating NSCs and could be harnessed to replace lost neurons, yet if overactivated it can hinder the later stages of neurogenesis, impeding neuroblast maturation and migration to the injury site when overexpressed (Gómez-Oliva et al., 2024).

ErbB2 is upregulated in injured regions and cooperates with NRG1 signaling to enhance NSC activation and neuroblast migration toward damaged tissue (Falnikar et al., 2018; Jia et al., 2021).

ErbB4 plays a central role in directing NSC migration and differentiation after brain injury (Kataria et al., 2019; Tagliaferro and Ponti, 2023). In both ischemia and TBI models, ErbB4 activation promotes neuroblast migration from the SVZ toward the injured cortex and supports their differentiation into mature, functional neurons. Recent findings show that NRG1 is primarily expressed by microglia at the injury site, and its increased availability facilitates the migration of SVZ-derived neuroblasts across the corpus callosum into the perilesional cortex, where they undergo progressive neuronal differentiation and synaptic integration, ultimately forming mature neurons (Pardillo-Díaz et al., 2025). Moreover, an *in vitro* study shows that NRG1 promotes neuronal over glial differentiation of NPCs, further underscoring its neurogenic bias when acting via ErbB4 (Domínguez-García et al., 2020).

Recent findings also show that TBI can activate proliferative responses beyond classical niches. Subcortical regions such as the striatum and optic tract exhibit trauma-induced neurogenesis and oligodendrogenesis, suggesting a broader regenerative capacity that may also be influenced by ErbB-related mechanisms (Astakhova et al., 2025).

### Interaction between notch and ErbB pathways to facilitate brain injury repair

Notch signaling plays a central role in the regulation of repairing mechanisms: neurogenesis, neuronal differentiation, and maturation (Zhang et al., 2018c). This highly conserved pathway influences cell fate decisions during embryonic development (Tiberi et al., 2012) and continues regulating NSC populations in the adult brain preventing premature differentiation, ensuring a reservoir of NSCs for brain maintenance and repair (Aguirre et al., 2010). In neurogenic niches, Notch signaling participates in the transition of NSCs to NPCs preventing the depletion of the NSC pool by inhibiting differentiation until the right signals are received (Gozlan and Sprinzak, 2023). Additionally, it influences the differentiation of NPCs into neurons, astrocytes, or oligodendrocytes as required (Guo et al., 2023; Gao et al., 2025). Beyond neurogenesis, Notch is also involved in neuronal maturation, guiding processes such as axon growth, synaptic formation, and plasticity. Disruptions in Notch signaling affect cortical neurogenesis and are linked to developmental disorders (Fiddes et al., 2018). On the contrary, its aberrant reactivation in adulthood is associated with neurodegenerative diseases, where it may contribute to gliosis and impaired neurogenesis (Zhang et al., 2018c).

Notch signaling is also important in the brain’s response to injuries, interacting with the ErbB family to regulate repair mechanisms (**Figure 5B**; Aguirre et al., 2010). In response to TBI, both Notch and ErbB1 pathways are activated in the SVZ and SGZ (Romero-Grimaldi et al., 2011; Wei et al., 2021). ErbB1 upregulation promotes NPC proliferation, while Notch signaling modulates their differentiation. Excessive ErbB1 activity can drive gliosis, limiting neurorepair. Notch ensures a balance by preventing premature glial differentiation, directing NPCs to differentiate into neurons or glial cells as needed, counterbalancing and promoting functional recovery (Scafidi et al., 2014; Gómez-Oliva et al., 2023a; Simpson Ragdale et al., 2023). The NPC migration towards the injured cortical areas, driven by ErbB signaling, is also regulated by Notch. Effective neurorepair relies on the balance between ErbB1-driven proliferation and Notch-mediated differentiation (Gómez-Oliva et al., 2023a; Simpson Ragdale et al., 2023). Similarly, in ischemic stroke, Notch-ErbB interaction regulates the balance between NPC proliferation and differentiation. However, an imbalance favoring ErbB1 can result in excessive gliogenesis, impeding neurogenesis, migration, and tissue repair (Aguirre et al., 2010; Piovesana et al., 2022; Simpson Ragdale et al., 2023; Gómez-Oliva et al., 2024). Although this review focuses on TBI and ischemic injury, it is worth noting that the interplay between Notch and ErbB1 signaling also contributes to neurodegenerative diseases such as Alzheimer’s disease. In these conditions, chronic neuroinflammation and amyloid-beta accumulation are associated with the reactivation of these pathways in NPCs and astrocytes, leading to altered neurogenesis and glial scarring (Tavassoly et al., 2020; Jayaswamy et al., 2023). Understanding how these pathways function in acute injuries such as TBI and ischemia could provide insights into therapeutic targets for chronic neurodegenerative conditions as well.

## Therapeutic Potential of Targeting ErbB Signaling to Repair Brain Injuries

Brain injuries can lead to lasting disabilities, cognitive and motor impairments, and increased dependency on care, affecting patients, families, and healthcare systems. Current pharmacological therapies focus on mitigating damage and managing systemic effects but cannot repair neuronal loss. For ischemic stroke, clot removal is achieved through thrombolytic drugs (e.g., tissue plasminogen activator) or mechanical extraction, while TBI and hemorrhagic stroke often require neurosurgery to reduce intracranial pressure. These treatments are effective only within narrow time windows and primarily prevent further damage. Rehabilitation, occupational therapy, and symptom management play critical roles in improving quality of life, with palliative care needed in severe cases (Tsai et al., 2024).

Despite ongoing scientific efforts to develop efficient therapies to repair brain injuries, no successful treatments have been developed to date. One promising avenue that we will late refer to as “strategy 1” involves reducing the exacerbated neuroinflammation caused by the hyperactivation of microglia and astrogliosis, thus preventing the formation of the glial scar through ErbB1 inhibition (Erschbamer et al., 2007; Li et al., 2011, 2014; Yang et al., 2011; Abrams et al., 2012; Gómez-Oliva et al., 2023a; Wang et al., 2023). This therapeutic approach could also be relevant for other CNS disorders, such as neurodegenerative diseases (Han et al., 2019; Mòdol-Caballero et al., 2020b; Tavassoly et al., 2020) where similar processes have been described. Alternatively, research has focused on taking advantage of the effects of the injury on neurogenesis and promoting migration of newly generated neurons towards the injury as well as their survival and maturation to replace the lost neurons with newly functional ones. This strategy focuses on either facilitating the release of ligands such as NRG1 and the activation of its receptor ErbB4 (Cespedes et al., 2018) or alternatively, avoiding excessive EGFR stimulation through inhibition of TGF-α release. We will later refer to this strategy as “strategy 2.” However, before explaining how these two strategies work, it is important to understand the different molecules that are able to mediate both inhibition and activation of ErbB receptors and that may be used for either strategy.

### ErbB receptor inhibitors used to promote neurorepair

One of the most compelling aspects of the ErbB receptor family is that numerous drugs targeting the inhibition of one or more ErbB receptors are already available on the market for the treatment of different types of cancer (Mòdol-Caballero et al., 2020a, b). Although these drugs are currently used in oncology, their existing approval could potentially facilitate their application in other diseases, as ErbB1 inhibitors have already undergone extensive clinical trials demonstrating their safety and efficacy. Moreover, some of these drugs have already been tested for CNS injury and spinal cord injury regeneration *in vitro* and in animal models (Abrams et al., 2012; Kjell et al., 2014; Scafidi et al., 2014; Hendry et al., 2016; Gómez-Oliva et al., 2023a). These drugs are mainly categorized into two groups: (a) Tyrosine kinase inhibitors (TKIs), which are small molecules that bind either reversibly or irreversibly to the intracellular catalytic domain of ErbB1. This inhibits the signaling cascade by blocking tyrosine trans-phosphorylation, preventing ligand-induced ErbB1 activation. Many of these inhibitors are specific to mutations found in cancer (Goldstein et al., 2020; Tsang et al., 2020; Cheng et al., 2023; Wang et al., 2024). (b) Monoclonal antibodies, which target the extracellular domain, either by blocking ligand binding or preventing receptor dimerization (Moradi-Kalbolandi et al., 2018; Sharifi et al., 2021; Habibi et al., 2025).

### Positive regulators of ErbB receptors

**Figure 6:**
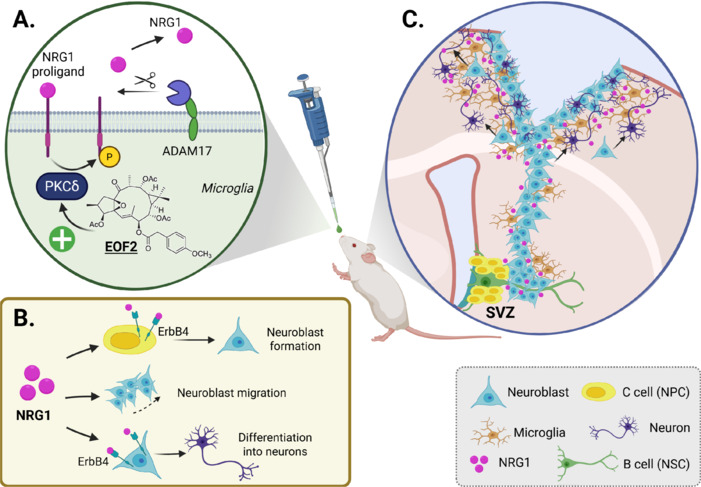
Mechanism of action of diterpene EOF2 and its role in cortical injury repair. (A) EOF2, a diterpene with a lathyrane skeleton, stimulates novel protein kinase C (PKC) isozymes, which in turn phosphorylate the transmembrane pro-ligand neuregulin-1 (NRG1). Once phosphorylated, the metalloprotease ADAM17 recognizes NRG1, catalyzing its cleavage to release the soluble form of NRG1. The soluble NRG1 can then bind to its receptor, ErbB4. As demonstrated in Pardillo-Díaz et al. (2025), this process occurs on the membrane of microglial cells. (B) NRG1 plays three key roles in cortical injury repair: it binds to ErbB4 receptors expressed in neural progenitor cells (NPCs) to promote neuroblast formation, acts as a chemoattractant to stimulate neuroblast migration, and, through ErbB4 signaling in neuroblasts, facilitates their differentiation into neurons. (C) Intranasal treatment with EOF2 following cortical injury (traumatic brain injury) has been shown to effectively enhance neurogenesis in subventricular zone (SVZ), promote the migration of neuroblasts to the injury site and improve the functionality of newly generated neurons contributing to effective neuronal repair. Created with BioRender.com.

Beyond these two groups of inhibitors, recent reports have highlighted the role of diterpenes with 12-deoxyphorbol and lathyrane structure as positive regulators of ErbB receptors (Geribaldi-Doldán et al., 2016; Murillo-Carretero et al., 2017; Saoudi Gonzalez et al., 2022). These molecules facilitate the release of ErbB ligands such as TGF-α and NRG1 (Geribaldi-Doldán et al., 2016; Domínguez-García et al., 2020; Pardillo-Díaz et al., 2025). These molecules specifically activate classical or novel protein kinase C isozymes resulting in the release of TGF-α (Domínguez-García et al., 2021; Gómez-Oliva et al., 2023b) or NRG1 (Domínguez-García et al., 2020; Pardillo-Díaz et al., 2025). As illustrated in **Figure 6A**, and as we have mentioned above, ErbB ligands are expressed in cells as membrane-bound pro-ligands, with their soluble domain facing the extracellular medium. The proteolysis of this domain, catalyzed by metalloproteases of the ADAM family, mainly ADAM17, results in the release of the soluble fragment (Dang et al., 2013). These metalloproteases select substrates previously phosphorylated by PKC isozymes (Dang et al., 2013). Each subfamily of PKC isozymes phosphorylates a group of ligands facilitating their release (Kamezaki et al., 2016). Recent reports have shown that diterpenes have the capacity to specifically activate a PKC isozyme, particularly diterpenes with 12-deoxyhorbol structure phosphorylate TGF-α through activating classical PKCα (Ezzanad et al., 2021; Gómez-Oliva et al., 2023b) and certain diterpenes with lathyrane structure stimulate the phosphorylation of NRG1 (Domínguez-García et al., 2020), as depicted in **Figure 6A**, thus facilitating its release. The use of classical PKC activating diterpenes activates Erk1/2 (Geribaldi-Doldán et al., 2016; Gómez-Oliva et al., 2023b) and cyclin D expression through EGFR signaling. Likewise, they promote hippocampal neurogenesis, NSC activation and proliferation *in vitro* (Geribaldi-Doldán et al., 2016; Domínguez-García et al., 2021) and *in vivo* in murine models not only stimulating cognitive function (Domínguez-García et al., 2021) but partially preventing cognitive decline in a murine model of accelerated aging (Gómez-Oliva et al., 2023b). Whereas the activation of EGFR by TGF-α may be beneficial at stimulating hippocampal neurogenesis, an excessive EGFR activity post brain injury is detrimental to neuronal replacement in the injured area (Romero-Grimaldi et al., 2011). Furthermore, the use of EGFR or ADAM17 inhibitors locally in injuries results in new neuron enrichment within the damaged area (Geribaldi-Doldán et al., 2018; Gómez-Oliva et al., 2023a). Comparably and as shown in **Figure 6B** and **C**, the use of novel PKC-activating diterpenes such as diterpene EOF2 promotes neurogenesis in the SVZ in response to injuries (Domínguez-García et al., 2020) facilitating the migration of neuroblasts toward cortical injured brain regions, which become mature functional neurons with specific features of cortical neurons (Pardillo-Díaz et al., 2025).

Besides, significant progress is being made in developing gene therapies targeting ErbB receptors. Promising strategies include siRNA, microRNA, RNA aptamers, synthetic transcription factors, and vitamin E isomers to regulate ErbB signaling (Chai et al., 2022). Cutting-edge therapies include CAR-T and CAR-M immunotherapies, which combine antibody specificity with T-cell or macrophage signaling, and two clinical trials targeting ErbB receptors are underway (Swain et al., 2023).

### Strategy 1: Modulating neuroinflammation through ErbB inhibition

The goal of this strategy is to modulate inflammatory signals released by overactivated microglial cells and astrocytes in response to damage in the case of TBI (Gómez-Oliva et al., 2023a), after ischemia (Yang et al., 2011), or after spinal cord injury (Erschbamer et al., 2007; Li et al., 2011, 2014; Abrams et al., 2012; Zhang et al., 2016).

The anti-inflammatory effects of afatinib have already been studied *in vitro* (Chen et al., 2019). A recent study from our laboratory (Gómez-Oliva et al., 2023a) hypothesized that inflammatory signals in response to injury, while increasing proliferation, might also prevent newly produced cells in the SVZ from maturing and migrating toward the lesion. In this study, the TKI afatinib was used to block the signal of one of the ErbB1 natural ligands, TGF-α, which is overexpressed by microglia *in vivo*. Afatinib facilitated the migration of neuroblasts from the SVZ to the lesion site, increasing their number in the perilesional area. This increase in neuroblasts in the perilesional area contributed to the repair of damaged tissue. The effect of blocking ErbB1 has also been studied in the context of ischemic stroke, specifically using the rat middle cerebral artery occlusion model (Yang et al., 2011). In the study by Yang et al. (2011), the inhibition of ErbB1 using a monoclonal antibody (C255, delivered to the lateral ventricle) suppressed the reactive astrogliosis triggered by middle cerebral artery occlusion and its associated neural injuries, significantly improving animal recovery. The beneficial effects of ErbB1 inhibition have also been seen in animal models of AD and ASL (Tavassoly et al., 2020), as well as in a model of peripheral nerve injury (Hendry et al., 2016).

However, the most used model to study ErbB1 inhibition after injury is undoubtedly the spinal cord injury. The lack of axon regeneration in the adult CNS following spinal cord injury is partly due to myelin inhibitors and the inhibitory properties of astrocytes (Erschbamer et al., 2007). In spinal cord injury, reactive astrogliosis not only forms a physical barrier to regenerating axons but also secretes molecules, such as chondroitin sulfate proteoglycans, which inhibit nerve growth (Li et al., 2011). Thus, it has been hypothesized that inhibiting ErbB1 could reduce neuroinflammation, suppress chondroitin sulfate proteoglycans production, and facilitate axonal regeneration after spinal cord injury (Li et al., 2011). Most studies use TKIs such as erlotinib (Kjell et al., 2014), imatinib (Abrams et al., 2012), and other potent non-commercial inhibitors such as PD168393 (Erschbamer et al., 2007; Li et al., 2014; Zhang et al., 2016b) or AG1748 (Li et al., 2011). Treatment with erlotinib (Kjell et al., 2014) showed accelerated locomotor recovery and slightly improved bladder function in the initial two weeks post-injury, although no long-term improvements in locomotor function were observed. Oral administration of erlotinib modestly enhanced the recovery rate, but the overall recovery outcome remained unchanged compared to untreated animals. In contrast, oral imatinib (Abrams et al., 2012), significantly improved blood-spinal cord-barrier integrity, locomotor and sensorimotor functions, and bladder recovery. Imatinib also reduced astrogliosis, chondroitin sulfate proteoglycans deposition, and inflammation, with these neuroprotective effects likely limiting secondary damage such as edema and hemorrhage. The irreversible ErbB1 inhibitor PD168393 (Erschbamer et al., 2007; Li et al., 2014; Zhang et al., 2016b) demonstrated more robust results. It significantly increased myelin basic protein expression, promoted glial precursor cell proliferation, and reduced apoptosis in the injured spinal cord (Zhang et al., 2016b). Intrathecal administration of PD168393 resulted in marked functional recovery, improving hindlimb motor function, bladder control, sensory function, and tissue sparing (Erschbamer et al., 2007). PD168393 also suppressed reactive astrogliosis, proinflammatory cytokine secretion, and chondroitin sulfate proteoglycans production, correlating with reduced demyelination and neuronal loss (Li et al., 2014). Similarly, AG1478 (Li et al., 2011) delivered locally with minipumps, reduced astrogliosis and chondroitin sulfate proteoglycans accumulation, while also increasing the expression of growth-associated protein-43, promoting a more favorable environment for axonal regeneration and improved functional outcomes in spinal cord injury models. The same effect was achieved with the monoclonal antibody C255 (Qu et al., 2012). Both AG1478 and C555 administered locally reduced cytokine production by inhibiting the ErbB1/MAPK cascade and depressing the activation of Erk and p38, as well as the interleukin-1 beta and tumor necrosis factor-alpha. This reduced microglia and astroglia activation, decreased tissue edema, and improved morphological and functional recovery after spinal cord injury.

Nevertheless, in addition to the studies demonstrating the potential of ErbB1 inhibition, there are also studies showing the protective role of EGF treatment (Justicia and Planas, 1999; Amin et al., 2010; Cooke et al., 2011; Scafidi et al., 2014). For example, in a study on hypoxia-induced neonatal brain injury, intranasal treatment with HB-EGF led to increased oligodendrocyte proliferation and survival, enhanced myelination, promoted neurogenesis, reduced apoptosis, modulated inflammation, and restored the blood-brain barrier. In contrast, treatment with gefitinib produced the opposite effect (Scafidi et al., 2014). These findings may be explained by the essential role of ErbB1 in neurogenesis and gliogenesis during development. ErbB1 expression is widespread across brain regions in the perinatal period, in contrast to the adult brain, where it is only found in NSCs and NPCs and is overexpressed in microglia and astrocytes following injury (Tavassoly et al., 2020). Additionally, various studies have linked ErbB1 activation to increased proliferation and survival of neural precursor cells, as well as reduced ischemic brain damage (Justicia and Planas, 1999). In a study by Cooke et al. (2011), a polymeric drug delivery system was used to administer EGF to the SVZ, which minimally invasively increased NSCs and NPCs proliferation by placing the system on the cortical surface. This system was proposed as a potential treatment for ischemic stroke (Cooke et al., 2011). However, while this delivery method seems promising for brain-targeted therapies, using a different ErbB1 ligand, such as NRG1, might be a better alternative, as it could promote proliferation without the negative inflammatory effects of EGF.

### Strategy 2: Promoting neuroprotection and neurogenesis through NRG1 release

NRG1 has been extensively studied in chronic heart failure, showing both efficacy and safety in clinical trials (Noll et al., 2024). A modified version, Neucardin, designed to preferentially activate ErbB4 homodimers and avoid cancer cell signaling, has been tested in Phase II trials (NCT01251406) (Cespedes et al., 2018). Additionally, NRG1/cimaglermin, the extracellular domain of NRG1β3, activates both ErbB4 and ErbB2 and has shown cardioprotective effects in models of treatment-induced heart pathologies (Cespedes et al., 2018). These advancements have led to its investigation in animal models as a potential treatment for brain stroke (reviewed in Cespedes et al., 2018; Noll et al., 2024). NRG1, administered within a window of up to 12 hours post-ischemia, significantly reduces infarct expansion into the penumbra, decreases ischemia-induced apoptosis, and restores BBB integrity, thus limiting edema and immune cell infiltration. It also promotes neurological recovery by enhancing axonal sprouting and synapse formation, with cimaglermin (NRG1β) being a potential candidate for stroke treatment (Cespedes et al., 2018; Noll et al., 2024). In stroke, NRG1 exerts its protective effects through the PI3K/Akt pathway (Noll et al., 2024). It also displays anti-inflammatory properties, preventing the activation of microglia, astrogliosis, and the expression of pro-inflammatory genes, contributing to improved neurological outcomes after stroke (Noll et al., 2024). In addition to ischemic stroke models, NRG1 has shown benefits in subarachnoid hemorrhage, where it improved BBB integrity, reduced neuronal injury, and ameliorated neurological deficits (Noll et al., 2024). Moreover, a previous study with heterozygous NRG1 knockout mice (NRG1^+/−^) and ErbB4 knockout mice revealed that the absence of these proteins leads to greater susceptibility to ischemic damage, highlighting the critical role of NRG1 in neuroprotection (Noll et al., 2024). Recent reports demonstrate that the diterpene EOF2, administered intranasally, facilitates the release of NRG1 at sites of cortical brain injury (Domínguez-García et al., 2020; Pardillo-Díaz et al., 2025). Using a murine model of mechanical lesions in the primary motor cortex to mimic TBI, we investigated the ability of EOF2-NRG1 pathway to enhance neuroblast migration and neuronal differentiation. EOF2 treatment promoted the migration of neuroblasts from the SVZ to the injury site and significantly enhanced their differentiation into mature, functional neurons (**Figure 6C**; Pardillo-Díaz et al., 2025). In the spinal cord injury model, it has been shown that following the insult, there is an acute and permanent depletion of neuronally derived NRG1 in the spinal cord, and that restoring normal levels through intrathecal administration of NRG1 enhances cell replacement and protects white matter, improving recovery (Alizadeh et al., 2018). Furthermore, in this same model, it was observed that NRG1 promotes a pro-regenerative immune response by stimulating a regulatory phenotype in T and B cells and increasing the population of M2 macrophages at the injury site. This inflammatory response was also balanced towards the production of pro-regenerative mediators over pro-inflammatory ones (Alizadeh et al., 2018). NRG1 has also been shown to improve the repair of myelin by Schwann cells in a model of Charcot-Marie-Tooth disease, promoting nerve repair and enhancing muscle function (Fledrich et al., 2014). Finally, NRG1 is gaining attention in the development of gene therapies, particularly in the SOD1 lateral amyotrophic sclerosis mouse model, as it is crucial for the survival of motor neurons primarily affected by this disease. Research indicates that the NRG1-ErbB4 pathway is impaired in individuals with lateral amyotrophic sclerosis (Mòdol-Caballero et al., 2020b). Various studies have utilized viral-mediated delivery of the NRG1-I (Mòdol-Caballero et al., 2020a) and NRG1-III (Lasiene et al., 2016) isoforms, showing promising results. Continuous expression of these therapeutic genes has led to functional improvements in skeletal muscles, enhancing collateral sprouting of motor axons (Mòdol-Caballero et al., 2020b) and restoring C-boutons (Lasiene et al., 2016). These effects are mediated by protection against excitotoxicity and inflammation, as well as the activation of cell survival pathways in both muscle and spinal cord (Mòdol-Caballero et al., 2020b). However, despite these promising findings, another study shows that intrathecal treatment with a NRG1 antagonist (HBD-S-H4) prevents microglial activation and motor neuron loss in SOD1 mice, leading to delayed disease onset and increased survival (Liu et al., 2018).

Together, these findings, summarized in **Table 1**, highlight the complexity and context-dependent nature of ErbB signaling in neural repair and inflammation. Both inhibition and activation of specific receptors or ligands may be beneficial depending on the type of injury and the therapeutic approach. While the existence of conflicting results underscores the need for further research, it also opens avenues for exploration, as understanding the precise role of ErbB in recovery could lead to targeted and effective therapies. These potential breakthroughs hold great promise for advancing the clinical translation of this pathway, ultimately improving outcomes for patients with diverse brain injuries.

**Table 1:** Summary of ErbB-related molecules with reported effects in CNS injury models

Molecule	Strategy	Effect	Outcome	Target	Model of injury	Reference
Afatinib	1	Indirect (TGF-α inhibition)	↑ Neuroblast migration, ↑ perilesional neurogenesis, ↓ inflammation	ErbB1	*In vitro*	Chen et al., 2019
					TBI	Gómez-Oliva et al., 2023a
Erlotinib	1	Direct	↓ Inflammation, modest functional recovery (early phase)	ErbB1	Spinal cord injury	Kjell et al., 2014
Imatinib	1	Direct	↑ Locomotor/sensorimotor recovery, ↓ astrogliosis, ↓ CSPGs, ↓ edema	ErbB1	Spinal cord injury	Abrams et al., 2012
PD168393	1	Direct	↑ Myelination, ↓ apoptosis, ↑ functional recovery, ↓ proinflammatory cytokines	ErbB1	Spinal cord injury	Erschbamer et al., 2007; Li et al., 2014; Zhang et al., 2016b
AG1478	1	Direct	↓ Astrogliosis, ↓ CSPGs, ↑ growth-associated protein-43, ↓ cytokines, ↑ axonal regeneration	ErbB1	Spinal cord injury	Li et al., 2011
C255 (antibody)	1	Direct	↓ Reactive gliosis, ↓ cytokines, ↑ recovery	ErbB1	Stroke (MCAO)	Yang et al., 2011
					Spinal cord injury	Qu et al., 2012
Gefitinib	1	Direct	↓ Neurogenesis, opposite of EGF effect	ErbB1	Neonatal hypoxia	Scafidi et al., 2014
HB-EGF	1	Direct	↑ Myelination, ↑ neurogenesis, ↓ apoptosis, ↓ inflammation	ErbB1	Neonatal brain injury	Scafidi et al., 2014
EGF	2	Direct	↑ Neurogenesis, ↑ oligodendrocyte survival, ↓ apoptosis, ↑ BBB integrity	ErbB1	Neonatal hypoxia	Justicia and Planas, 1999
					Ischemia	Amin et al., 2010; Cooke et al., 2011
NRG1 (general)	2	Direct	↓ Apoptosis, ↓ inflammation, ↑ synaptogenesis, ↑ neurogenesis, ↑ functional recovery	ErbB4/ErbB2	Ischemic stroke	Cespedes et al., 2018; Noll et al., 2024
					SAH	Noll et al., 2024
					SCI	Alizadeh et al., 2018; Noll et al., 2024
					Charcot-Marie-Tooth disease	Fledrich et al., 2014
					ALS	Mòdol-Caballero et al., 2020b
Cimaglermin (NRG1β)	2	Direct	↓ Infarct volume, ↑ neurological recovery, BBB restoration	ErbB4/ErbB2	Stroke	Cespedes et al., 2018; Noll et al., 2024
EOF2	2	Indirect (NRG1 release)	↑ neuroblast migration, ↑ differentiation	ErbB4	TBI	Pardillo-Díaz et al., 2025
NRG1 (intrathecal)	2	Direct	↑ White matter protection, ↑ immune modulation (M2), ↑ recovery	ErbB4	Spinal cord injury	Alizadeh et al., 2018
NRG1 (gene therapy)	2	Direct	↑ Motor neuron survival, ↑ axonal sprouting, ↓ excitotoxicity, ↓ inflammation	ErbB4	ALS	Lasiene et al., 2016
HBD-S-H4 (NRG1 antagonist)	2	Direct	↓ Microglial activation, ↓ motor neuron loss, ↑ survival	ErbB4	ALS	Liu et al., 2018

The column “Strategy” refers to the therapeutic approach explored: Strategy 1 focuses on reducing neuroinflammation and preventing glial scar formation. Strategy 2 aims to promote endogenous repair mechanisms. The column “Effect” indicates whether the molecule acts directly on an ErbB receptor (e.g., as a ligand or receptor inhibitor) or indirectly by modulating upstream or downstream elements that influence ErbB activity. ALS: Amyotrophic lateral sclerosis; BBB: blood-brain barrier; CNS: central nervous system; CSPGs: chondroitin sulfate proteoglycans; EGF: epidermal growth factor; EOF2: 3,8,12-tri-O-acetyl-7-O-(4-methoxyphenyl) acetylingol, CAS number 2230806–06–9; HB-EGF: heparin-binding EGF-like growth factor; MCAO: middle cerebral artery occlusion; NRG1: neuregulin 1; SAH: subarachnoid hemorrhage; SCI: spinal cord injury; TBI: traumatic brain injury; TGF-α: transforming growth factor alpha.

## Conclusion

ErbB receptors play a critical role in the intricate processes of brain repair, including neurogenesis, gliogenesis, and synaptic plasticity. The pathways initiated by these receptors either alone or by interacting with other signaling cascades modulate glial scar formation, neuroinflammation, neuronal survival, and neural tissue repair making these receptors and their ligands suitable targets for developing therapeutical strategies to treat brain injuries. We discussed here two possible pharmacological strategies involving ErbB receptors: a first strategy directed to prevent neuroinflammation through ErbB inhibition and a second strategy directed to promote neuroprotection and neurogenesis through the use of NRG1 or the facilitation of NRG1 release.

ErbB inhibitors, such as afatinib, are currently approved and in clinical use for the treatment of certain cancers, where their ability to block aberrant ErbB signaling has proven beneficial. Interestingly, recent findings suggest that these compounds may exert additional effects beyond tumor suppression, notably by attenuating neuroinflammation. This opens the door to an exciting therapeutic possibility: repurposing ErbB inhibitors to promote recovery following brain injury. By dampening inflammatory responses and potentially modulating regenerative pathways, these agents could be leveraged in a new context—transforming a class of drugs originally developed for oncology into promising tools for neural repair. Exploring this avenue may not only accelerate drug development by building on existing safety profiles, but also offer novel strategies to harness the plasticity of the injured brain.

Recent findings point to diterpenes as promising natural modulators of neuroregenerative signaling pathways, with the potential to enhance neurogenesis and support brain repair. Although the precise mechanisms through which these compounds interact with ErbB receptors are still being elucidated, their therapeutic promise is increasingly evident. Uncovering the molecular crosstalk between diterpenes and ErbB signaling could open new avenues for intervention, particularly in the context of neurodegenerative diseases. Future efforts should prioritize dissecting these interactions and advancing the translational potential of diterpenes through rigorous preclinical and clinical studies. Deepening our understanding of this interplay may ultimately lead to innovative, nature-inspired strategies to restore brain function.

## Data Availability

*Not applicable*.
